# Beyond the food on your plate: Investigating sources of microplastic contamination in home kitchens

**DOI:** 10.1016/j.heliyon.2024.e35022

**Published:** 2024-07-24

**Authors:** Vilde K. Snekkevik, Matthew Cole, Alessio Gomiero, Marte Haave, Farhan R. Khan, Amy L. Lusher

**Affiliations:** a Norwegian Institute for Water Research (NIVA), Oslo, Norway; b Marine Ecology & Biodiversity, Plymouth Marine Laboratory (PML), Plymouth, PL1 3DH, UK; c Norwegian Research Centre (NORCE), Department of Climate & Environment, Mekjarvik 12, 4072, Randaberg, Norway; d SALT Lofoten AS, Pb. 91, Fiskergata 23, 8301, Svolvær, Norway; e Norwegian Research Centre (NORCE), Department of Climate & Environment, Nygårdsgt 112, 5008, Bergen, Norway

**Keywords:** Microplastics, Food preparation, Food safety, Kitchen equipment, Utensils, Microplastic release mechanisms

## Abstract

Given that a substantial amount of time is spent in kitchens preparing food, the kitchen equipment used may be relevant in determining the composition and amount of microplastics ending up on our dinner plate. While previous research has predominantly focused on foodstuffs as a source of microplastics, we emphasise that micro- and nanoplastics are ubiquitous and likely originate from diverse sources. To address the existing knowledge gap regarding additional sources contributing to microplastics on our dinner plates, this review investigates various kitchen processes, utensils and equipment (excluding single-use items and foodstuffs) to get a better understanding of potential microplastic sources within a home kitchen. Conducting a narrative literature review using terms related to kitchenware and kitchen-affiliated equipment and processes, this study underscores that the selection of preparation tools, storage, serving, cooking, and cleaning procedures in our kitchens may have a significant impact on microplastic exposure. Mechanical, physical, and chemical processes occurring during food preparation contribute to the release of microplastic particles, challenging the assumption that exposure to microplastics in food is solely tied to food products or packaging. This review highlights diverse sources of microplastics in home kitchens, posing concerns for food safety and human health.

## 1 Introduction

Microplastics (usually defined as plastic particles between 0.1 μm and 5 mm [1]) have been found in every environment that has been studied (e.g. Refs. [[2], [3], [4]]). Microplastics can originate from the breakdown of larger plastics (e.g., from packaging, fishery and agriculture activities, tyres etc. [5]) via chemical, physical or biological processes [6,7] or by being intentionally manufactured at microscopic level [[8], [9], [10]]. Fragmentation of plastics can also result in the formation of nanoplastics (<0.1 μm) [11]. Ecotoxicological studies have demonstrated microplastics can be readily consumed and translocated into blood and organs, possibly causing and cause adverse health effects in wildlife, inevitably leading to concerns for human health [12,13]. Humans can be exposed to micro- and nanoplastics via ingestion, inhalation, or dermal contact [[14], [15], [16]]. Ingestion may occur through the consumption of food and drinks contaminated with micro- and nanoplastics such as seafood or marine products (e.g., fish, shellfish, sea salt), drinking water, or beverages [12,17].

Micro- and nanoplastics may contaminate food products via numerous pathways. For example, microplastics may contaminate source material (e.g. ingestion of microplastics by animals or fish [18]); airborne contamination during processing [19,20]), from the packaging materials [21], or during food preparation [22]. The ingestion and inhalation of microplastics poses health concerns, including the potential for adverse effects on the digestive tract [23], reproductive organs [24], respiratory tract and other systems [14]. Understanding and addressing the different sources of micro- and nanoplastic contamination are important steps for being able to mitigate any potential effects.

To date, an extensive number of research papers have established the presence of micro- and nanoplastics in food items [12] however, home kitchens may serve as a significant contributor of human exposure to micro- and nanoplastic particles owing to the prevalence of furnishings, cookware, food contact materials and electrical goods manufactured from plastic [[25], [26], [27]]. The current understanding of how various cookware items might release micro- and nanoplastics is unclear. To address this critical knowledge gap, this review aims to explore the extent to which kitchenware, cooking and cleaning and equipment may release micro- and nanoplastics into the kitchen environment, resulting in human exposure. Particular attention is drawn to how different processes, such as abrasion between utensil and cookware, and heating of plastic ware, may affect the release of plastic particles. Notably, this narrative review does not consider the contribution of food or water, or single-use items (e.g. packaging) in contributing to human microplastic exposure, given these topics have been reviewed extensively [[28], [29], [30], [31], [32], [33], [34]].

In a two-step process, we firstly identified the items commonly used in home kitchens and then employed this list of items as search terms in a systematic search of the primary literature on micro- and nanoplastics. The risk of micro- and nanoplastic contamination is evaluated for individual items and processes, drawing out commonalities where evident. Owing to variations in analytical methods, reported size-classes, measurement units and contamination controls, it was not possible to make direct comparisons between the number of micro- and nanoplastic that might be released from given items or processes which makes result comparisons difficult [35].

## 2 Methods

### 2.1 Sourcing of literature material

**Fig. 1:**
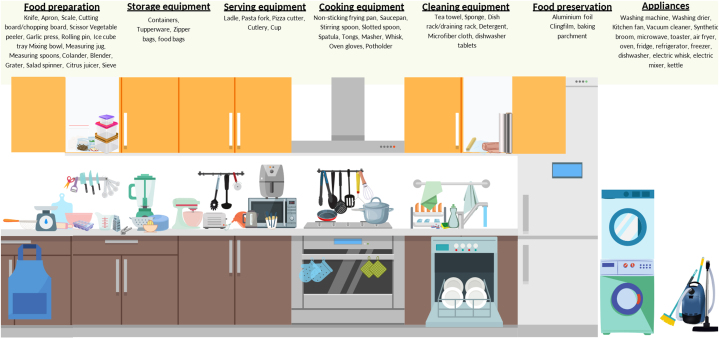
Illustration of kitchen categories and their associated equipment and tools. Infographic produced by using the free-to-use online graphic design tool canva.com.

To investigate the occurrence of microplastics in the home kitchen, including the multiple activities within the kitchen, we undertook a narrative review of the scientific literature. A search was performed using ISI Web of Knowledge/Web of Science in February 2023. This was complemented with additional searches on both ISI Web of Knowledge/Web of Science and Google Scholar in May 2023 and June 2024 to ensure comprehensive coverage of the available literature. Given the relatively low numbers of papers identified, the reference lists of relevant papers were scoured for any additional texts of high relevance to this review. Keywords used in the first search were based on the list of commonly used kitchen items typically used in a home kitchen was derived through peer discussion and online recommendations (e.g., Kitchen Essentials List [36]). This list was compiled and divided into seven broad categories (Fig. 1). The search terms comprised of: TS=(plastic* OR synthetic* OR microplastic* OR nanoplastic* AND (*kitchen utensils OR food preparation OR knife OR cutting board OR scissors OR vegetable peeler OR garlic press OR grater OR kitchen scale OR measuring jug OR measuring spoon OR mixing bowl OR colander OR rolling pin OR blender OR salad spinner OR citrus juices OR ice cube tray OR apron OR non-sticking frying pan OR saucepan OR stirring spoon OR slotted spoon OR spatula OR tongs OR master OR whisk OR oven gloves OR pot holder OR kettle OR ladle OR pasta fork OR pizza cutter OR cutlery OR cup OR tea towels OR sponges OR dish rack OR detergent OR microfiber cloth OR food preservation OR clingfilm OR aluminium foil OR containers OR zipper bags OR Tupperware OR washing machine OR washing drier OR kitchen fan OR vacuum cleaner OR synthetic broom OR chopping board OR weighing scale OR sieve OR ice tray OR draining rack OR dishwasher tablets OR baking parchment OR food bags OR microwave OR toaster OR kettle OR air fryer OR oven OR fridge OR refrigerator OR freezer OR dishwasher OR electric whisk OR electric mixer*)). Inclusion of laundry facilities was included given their commonality in home kitchens in certain countries (e.g. United Kingdom). Refined languages included Swedish, Norwegian, Danish, English, and unspecified language, and all articles from 1965 to 2024 were included in the search. The supplementary searches were performed to account for the duration of time from the first search. Only articles published up until June 2024 were included in the data analysis of this review. The relevance of the resultant articles was determined by reading through the title and abstract and subject to a quality control step (see below). Only literature that passed this stage was utilised in the review.

### 2.2 Quality control

To avoid overlap with other reviews, articles focussed upon drinking water, food or beverages, airborne particles, or single use items (e.g. takeaway containers and packaging materials) were excluded. While single-use plastics can be present in the kitchen, they're not typically considered as “kitchenware and appliances” which was the focus of this review. The primary literature obtained from the literature searches had to fit the following quality criteria to be included in this review. Firstly, the article had to have undergone peer-review prior to publication, ensuring a foundational level of quality assurance. Secondly, we exclusively selected articles that focused on items typically found in a kitchen (Fig. 1).

If any of these parameters were not met, the paper was not included in this review. Following screening, a total of 30 articles were selected to be included in this review. Next, articles were categorised into seven main categories (Fig. 1): 1) Food Preparation, 2) Storage Equipment, 3) Serving Equipment, 4) Cooking Equipment, 5) Cleaning Equipment, 6) Food Preservation, and 7) Appliances. The division of papers between categories is presented in Table 1. Papers were reviewed to ascertain the potential particle release and the underlying causes of fragmentation of plastic particles in home kitchens.

**Table 1:** Total numbers of papers identified in each category of kitchen processes describing the release of micro- and nanoplastics.

Category	Investigated kitchen utensil	Papers identified as relevant for this perspective/review
Food Preparation	Cutting boards	Habib et al., 2022a
	Chopping boards	Luo et al., 2022
	Cutting boards	Yadav et al., 2023
	Cutting boards	Habib et al., 2022b
	Mixing bowls	Jander et al., 2022
	Kitchen blender	Luo et al., 2023
	Chopping boards, PTFE-coated pans, jug, knife and spoon	Cole et al., 2024
Food Storage	Food container	Hee et al., 2022
	Food containers	Hussain et al., 2023
Cooking Equipment	Non-stick cookware	Luo et al., 2022
Cleaning Equipment	Cleaning sponge	Luo et al., 2022
Food Preservation	Victuals Packaging	Guan et al., 2021
Appliances	Kettle	Shi et al., 2022
	Washing machine	Belzagui et al., 2019
	Washing machine	Berruezo et al., 2021
	Washing machine	Cai et al., 2021
	Drying machine	O'Brien et al., 2020
	Washing machine	Dalla Fontana et al., 2021
	Washing machine	De Falco et al., 2020
	Washing machine	Dreillard et al., 2022
	Washing machine	Galvao et al., 2020
	Washing machine	Hartline et al., 2016
	Washing machine	Jenner et al., 2021
	Washing machine	Lant et al., 2022
	Washing machine	Lim et al., 2022
	Washing machine	Palacios-Marin et al., 2022
	Washing machine	Vassilenko et al., 2021

### 2.3 Caveats with the retrieved data

Statistical analysis was omitted from the data collected, as it was treated as absolute values without any efforts made to compare the particle release values due to variations in detection and analysis methods. Moreover, the lack of contamination control and reporting in some papers prevents precise estimations of particle counts, offering only insights into potential polymer types and cooking processes that might yield higher particle release compared to alternative materials and cooking techniques.

## 3 Potential sources of microplastic contamination in home kitchens

### 3.1 Food preparation

Mechanical stress and abrasion of plastics are known to generate microplastics and shifts in temperature and pH may also cause plastic degradation [6]. Food preparation activities involving mechanical stress include cutting, mixing, scraping, and whisking, and these processes are also thought to generate micro- and nanoplastics [37]. In kitchens, temperature fluctuations are frequent, including freezing (-20 °C), refrigeration (2 °C), washing up (40 °C), heating and sterilisation (100–250 °C). Heating plastic ware can impact the thermal integrity of the plastic product promoting the release of micro- and nanoplastics and chemical leachates within the product.

Of the various food preparation activities that occur in the kitchen, the mechanical stress on plastic cutting boards when in use has received a relatively large proportion of recent attention, though the actual number of studies is still low [25,26,37,38]. Yadav et al. [37] focused on three factors associated with the use of cutting boards – chopping style, cutting board material, and mimicking real-life application by slicing a carrot on the board. The release of microplastics (20–200 μm), was impacted by chopping style and different materials, and thousands of microplastics were estimated to be generated over six cycles of use. The use of polypropylene (PP) cutting boards generated three-four times as many microplastics compared to polyethylene cutting boards over six cycles of use, when controlling for chopping technique. The difference, however, was not statistically significant. Perhaps most interesting was that the chopping of a carrot on the PE cutting board produced almost three times more microplastics compared to use without the vegetable. This may be explained by the greater pressure required when cutting through the carrot leading to the knife penetrating deeper into the chopping board, thereby excising more microplastics. Extrapolating to an annual exposure per person, the authors suggested an exposure range of 7.4–50.7 g of microplastics from a single PE chopping board.

Cutting boards are not uniform especially after extensive use, and chopping techniques can alter micro- and nanoplastic generation. To mimic the cutting process and simplify the sample preparation, Luo et al. [38], sliced or scratched a knife on a chopping board surface, without food items. After each cutting action (per cut), the authors directly tested the released debris on the knife and on the chopping board (along the cutting groove). They estimated a release of 100–300 micro- and nanoplastics per mm per cut along the groove formed, and ∼3000 per mm^2^ per cut in the scratched area on the chopping board. This suggests that the pre-existing condition of the board is an important determinant in micro- and nanoplastics generation. The authors concede that the actual action of cutting food may alter the dynamics of microplastic formation as shown by Yadav et al. [37], but the study does highlight that cutting boards have zones in which cutting is more common and this affects the generation of micro- and nanoplastics. Such insight would be relevant for those cutting boards in constant use, such as those that are used in commercial settings, or boards maintained by households over sustained periods.

In Middle Eastern butchers and supermarkets, PE-based plastic cutting boards were identified as a source of micro- and nanoplastics in prepared meat and fish products [25,26]. Investigating the potential for cutting boards as a source of microplastic contamination in red meat (goat and beef), Habib et al. [25], showed boards contributed 1–7 microplastics per g meat, compared to 0 microplastics found when the meat was processed on a bamboo cutting board or with a metallic meat grinder. Scraping the residual meat from the plastic board at the end of day resulted in approximately 70 microplastics per g meat. In a follow-up study, investigating produce sourced from supermarkets where produce was cut on plastic boards, microplastics levels ranged from approximately 0 - 1 particle per g-1 chicken and from 0 to 2 particles per g-1 fish [26]. In chicken, the average microplastic size was around 100 μm whereas in fish the average microplastic size was 190 μm. Both studies reported that washing the meat before cooking effectively reduced microplastic loads but did not eliminate them entirely.

Mixing bowls are another aspect of food preparation that involve mechanical stress. Jander et al. [39], exposed six different bowls (acrylonitrile–butadiene–styrene (ABS), PP, melamine, PE, polystyrene (PS), and styrene–acrylonitrile (SAN) to a hand mixer for 2 min at 200 rpm. Microplastics were released into purified water as standard, but the ABS bowl was additionally subjected to mixing in the presence of rock salt in water to simulate a granular matrix. The results showed a slight difference between bowls of different materials. Abrasion caused by the salt granules increased microplastics release three-fold. In the second part of the study pyrolysis was performed, revealing several chemical components released at temperatures of 200 °C and 250 °C, which reflect commonly used cooking temperatures. All polymers released chemicals, but the results of the analysis of the melamine polymer was particularly concerning, given the release of formaldehyde, a known toxicant and carcinogen. Additionally, the age of cookware and kitchen utensils can significantly influence the amount of microplastics they release. In a study by Cole et al. [22], researchers investigated the effect of old and new kitchen utensils on the release of plastic particles. The study revealed that older plastic cookware, identified by signs such as staining, heat damage, scratches etc., released more microplastics compared to their newer and non-plastic alternatives.

Melamine and melamine–formaldehyde resin is widely used in many applications including kitchen utensils and camping crockery owing to their properties of hardness, heat resistance, and physical and chemical stability [40]. Kim et al. [41], investigated the migration of monomers (melamine and formaldehyde), plastic additives, and non-intentionally added substances (NIAS) from melamine–formaldehyde resin food utensils into food simulants after UV irradiation for up to 7 d. After UV exposure, the release of plastic additives increased dramatically in melamine-formaldehyde resin, which was not the case for the other polymers (PP, PA, PE, PET, and Silicone). The additives included plasticizers, slip agents, surface-active agents, and coating agents. The study highlighted that melamine–formaldehyde resin is less resistant to UV than other polymers. However, an overall evaluation of food safety based on US FDA guidelines of tolerable daily intake (TDI) and estimated daily intake (EDI) showed that the monomers presented a low risk, though care may be needed if prolonged UV sterilisation occurs (e.g., in an industrial kitchen).

The studies focusing on mechanical and thermal processes in the kitchen demonstrate pathways for microplastics and their chemical constituents to enter food items. For example, significant increases of heavy metal concentrations (aluminium, arsenic, cadmium, and lead) have been observed when boiling water for 1–4 h in a plastic kettle and PTFE-coated pan [42]. Further, a study conducted by Luo et al. [43], demonstrated blending an ice block for 30 s in a kitchen blender could release approximately 0.26-0.36 × 10^6^ microplastics. However, as this study highlights, it is important to acknowledge that the extent of the release is influenced by several factors, including the material composition of the blender container, rotational speed, and configuration settings, as well as the hardness, quantity, size, and temperature of the food being blended [43].

### 3.2 Storage equipment

Plastic storage containers and bags are commonly used in households for preserving food, given that they are airtight, waterproof, lightweight, and durable. Food containers will often include repurposed takeout containers given their design and availability. Recent studies have revealed a release of microplastics from plastic storage equipment, with significant variations based on storage conditions, food types, and cooking methods [34,44,45].

Material used to manufacture storage containers can also influence the quantity of microplastics discharged [34]. Polystyrene (PS) containers, due to the softer texture, low rigidity compression resistance, exhibit looser structures and coarser surfaces [46]. These features might explain why PS containers shed more microplastic than other types of plastic containers [34,47,48]. Notably, PS containers had physical defects such as surface cracks and gaps on the surface. Furthermore, the container's surface retained PS particles, likely originating from the manufacturing process [49]. The extent of microplastic release can also be influenced by factors such as contact area and friction between the food and the take-out container, as demonstrated in various studies focusing on lid mechanics in plastic bottle caps [50,51]. In summary, the quantity of microplastic release is influenced by choice of material and surface properties, where softer texture and structural characteristics can cause microplastic to enter our foods and be a source of microplastic being brought into our kitchen.

Exposure to high temperatures can cause plastics to degrade and fragment, potentially contaminating food with micro-and nanoplastics. The contamination may start when the raw materials are stored in plastic-based packaging at high temperatures, such as when reheating food in microwaves. At given polymer type-dependent thermal energy, a free radical process generation occurs with successive removal of lower molecular weight fragments from plastic storage via random and chain end degradation [52]. Release of micro- and nanoplastics of various shapes and sizes is highly likely to be due to the heat-induced flaking of the cracked plastic surfaces. Repeated heating of PET and PP packaging results in consistent leaching of plastic particles, suggesting that reheating food and beverages in such “reusable” plastic containers is a food safety risk. Hussain et al. [53], found PP containers heated in a microwave released significantly more microplastics compared with containers stored at room temperatures or in refrigerators. The study also tested the influence of deionized water (DI) (to simulate aqueous food) versus 3 % acidic acid (to simulate acidic food). Under refrigeration conditions the containers with DI-water released between 577 and 415 thousand microplastics/cm^2^ and under room-temperature conditions between 95 and 841 thousand/cm^2^. However, under microwave heating, the containers released between 425 and 4.22 million/cm^2^ microplastics, underscoring that increased temperature can cause an acceleration in polymer chain scission, and consequently increased release of microplastics. The difference between released number of particles between containers holding DI-water and 3 % acidic acid also demonstrates that overall, more particles were released during room-and refrigerator temperatures conditions in 3 % acidic acid. This highlights the potential influence of acidic food on particle release [53]. In addition to variable temperatures, chemical composition, and exposure time to food and drinks, we consider that varying material quality - stemming from differences in production quality and the age and degradation of a product - may also contribute to fluctuating degrees of wear and tear on plastic ware.

### 3.3 Serving equipment

While we identified several studies investigating the release of microplastics from drinking water bottles and feeding bottles for children [[54], [55], [56]], and single use plastic cups and cutlery [57,58], we found no research on the release of microplastics from plastic serving equipment such as ladles, pasta forks and pizza cutters. These serving utensils are in direct contact with our food and highlights the knowledge gap of potential sources of microplastics within our kitchens.

### 3.4 Cooking Equipment

Cooking in the kitchen utilises a variety of equipment and appliances, often subject to high temperatures. Non-stick pans, widely used in kitchens for their convenience, are designed to prevent food from sticking to the pan when cooking [59]. Non-stick pans, utensils and rice-cookers are coated in polytetrafluorethylene (PTFE), a polyfluorinated synthetic plastic material with both water and fat-repellent properties. Concerns have been raised about the potential health risks of non-stick cookware when they exceed certain temperatures (>350 °C) as they may release toxic fumes [60,61]. Additionally, it has been highlighted that micro- and nanoplastics might be formed if non-stick materials are abraded or scratched, for example when using utensils or scourers [62]. Luo et al. [62], identified that using a stainless-steel turner, BBQ clamp, stainless steel wool and word turners on new and old non-stick cookware all released PTFE particles. Their findings highlighted that a broken part of the Teflon could release 2,300,000 micro- and nanoplastics, while a fractured part from a scratch could release around 9100 micro- and nanoplastics. Additionally, a recent study identified that even using a soft silicone whisk with newly purchased non-stick pans could result in the release of PTFE particles [22]. Cooking processes may also affect particle release, with prolonged frying of food demonstrated to release more particles than when baking in the oven [34,44].

### 3.5 Cleaning Equipment

Cleaning equipment and items manufactured from plastic including sponges, tea towels, dish rack/draining racks, detergents, microfiber cloths and dishwasher tablets are commonly found and used in home kitchens. The mechanical process of cleaning, which involves friction between the cleaning item and the surface, can result in the degradation and shedding of microplastics [63]. These cleaning items come in direct contact with various surfaces such as countertops, dishes, and utensils, facilitating the contamination of food and human exposure to microplastics. The material composition of the cleaning item can influence the level of particle release [64]. For example, dish sponges can be manufactured from nylon, polyester (PES), PE, and cellulose [65]. In a study Luo et al. [66], examined the shedding of microplastics from sponges by mimicking the cleaning process using a soft and hard layered sponge on a glass surface to simulate smooth surfaces and knives and forks to simulate sharpen edges. The study revealed that nylon PA-6 particles were shed from the soft layer of the sponge, while PET particles were released from the hard side. Although this study focused on a specific type of sponge, the results give an indication that microplastics might be released in the cleaning process. While this study did not include detergents, we consider chemical detergents, containing surfactants, bleach and abrasives, may exacerbate the release of microplastics. Older sponges are likely to release particles more easily due to the breakdown of the core network on the sponge [66]. Even though no studies have been performed on microfiber cloths and the potential release of microplastic fibers due to the friction when cleaning, most microfiber cloths are composed of synthetic materials such as PES or PA, potentially shedding tiny plastic fibers [64,67,68].

### 3.6 Food preservation

Plastic coverings and preservation items, such as clingfilm and silicone wrap, have made a significant contribution to food preservation, by effectively shielding food from oxygen, water vapour and microorganisms [69]. These food preservation materials often play a significant role in our daily lives, whether it is for reheating leftovers in the microwave, covering food in the fridge or being used in baking. By being in direct contact with food products under different temperatures (ovens, refrigerators), these products and materials can be a potential source of micro-and nanoplastics into our food. On a general basis, to ensure the safety of these materials for human use, various regulatory measures have been implemented, though primarily focusing on the release of additives and chemicals associated with plastics [70], but not plastic particles. The release of micro- and nanoplastics from clingfilm, baking parchment and silicone wrap are poorly elucidated. We only identified one study on clingfilm (food wrap), which revealed that irregularly shaped PE particles were released from preservative film when exposed to deionized water at temperatures of 50 °C, 75 °C and 95° [46]. This suggests that release of micro- and nanoplastics may occur when these materials are subjected to high temperatures and highlights the need for further studies considering the frequent use of such materials in food preservation.

### 3.7 Appliances

Several appliances are typically found within a kitchen, e.g., kettles, microwaves, ovens, kitchen fans, vacuum cleaner, brooms, toaster, air fryer, fridge, dishwasher, freezer and electric whisk and mixer, and in some instances washing machines and dryers. Our systematic searches revealed that very few studies have explored release of microplastics from kitchen appliances. While a few studies have explored microplastic release from kettles, there is a significant lack of research on other commonly used kitchen appliances. Shi et al. [71], investigated the release of micro- and nanoplastics when boiling water in plastic kettles, revealing a notable decrease in the release of micro-and nanoplastics from the plastic kettle after an extended period of usage. The authors describe their finding as a natural passivation phenomenon, which confirms that the natural formation of films with diverse chemical compositions in kettles, influenced by the ions in local regional water supplies, affects the number of released micro- and nanoplastics by acting as a barrier to the release of particles.

While washing machines and dryers are not commonly considered to be kitchen appliances, in some countries (notably the United Kingdom) they are placed there. Although both appliances have been extensively examined as sources of micro- and nanoplastics, specifically microfibers, in the environment (via wastewater) [[72], [73], [74], [75], [76]], there is a noticeable gap in research regarding their potential contribution within a domestic setting. With regard to the contamination within the kitchen, laundry facilities, if placed there, may well contribute to the release of microfibers into the air, ultimately affecting human exposure via deposition onto kitchen surfaces Microfibers predominantly originate from textiles, whether natural or synthetic [72,73]. Research by Lim et al. [74], revealed that over 50 % of microfibers are released during washing and wearing, posing a substantial human exposure risk. Textiles in Western households encompass clothing, and dish cloths, hand towels, upholstery, curtains and carpets that might be present in domestic kitchens. Numerous studies have explored the release of microplastic microfibers during a textile's lifespan, often focusing on their presence in wash water rather than considering human exposure through inhalation or ingestion [67,75]. To date, few studies have observed direct microfiber release into the air from laundry [76] and its potential impact on indoor human exposure. Indoor exposure to microplastics is increasingly recognised as significant, given that people spend approximately 90 % of their time indoors [77]. Jenner et al. [78], reported substantial daily microplastic deposition rates indoors, with small fibrous particles (5–250 μm) being the most prevalent (90 %); this size of particles are small enough to be inhaled deep into the lungs where they have the potential to cause physiological harm. Dreillard et al. [79], found 40–75 % of microfibers were between 50 and 200 μm, with average microfiber diameters ranging from 8 to 17 μm which was similar to the findings by Galvão et al. [80], where 53 % of the microfibers were between 50 and 100 μm. Various factors of appliance efficiency, washing or drying conditions (e.g. temperatures) and fabric quality (e.g. chemical composition, textile surface density, yarn type and aging garments etc.) will influence the release of microfibers from washers and dryers [[73], [81], [82], [83], [84], [85], [86], [87], [88]], but ultimately placing dryers in kitchens and living areas may increase exposure risks through air and deposition on kitchen surfaces. Thus, relocating laundry facilities away from living spaces could be an effective strategy to reduce daily exposure to microplastics in the form of microfibers.

## 4 Conclusion

Given global usage of plastic, exposure to micro- and nanoplastics is inevitable. However, consideration of utensils, cookware and equipment usage and placement in the domestic settings may be useful in reducing exposure. While microplastics have been detected in a range of food items, with the underlying assumption that the microplastic comes from the food, packaging or airborne contamination, we highlight that the choice of preparation, storage, serving, cooking and cleaning materials in our kitchens are also substantial contributors. Mechanical, physical, and chemical processes are the predominant factors driving the generation of micro- and nanoplastics from plastic materials during food preparation. The release of micro- and nanoplastics may be influenced by myriad factors, including material quality, forces applied, temperature and material type, age and integrity. For example, exposing non-stick and plastic cookware to high temperatures can release toxic fumes, degradation of the plastic and migration of plastic leachates into food. Similarly, microplastics can be released from the inner surface of food containers during freezing and thawing cycles. The ageing and manufacturing processes of utensils can significantly influence the release of microplastics, with extended use and wear increasing the likelihood of malformations, cracking and oxidization of the plastic. With non-stick cookware and utensils prolonged used can result in the loss of protective layers or coatings, allowing for increased contact between the utensils and the food being prepared and loss of integrity. Cleaning equipment, such as sponges and cloths, can shed microfibers during the cleaning process, with evidence suggesting age and type of materials both influence deposition rates. Finally, kitchen appliances, such as washing machines and dryers located in the kitchen may increase the likelihood of airborne microplastic fibres.

## Funding

www.namc.noThe project was funded through the North Atlantic Microplastic Centre (www.namc.no) project. VKS and AL recieved additional financial support from the 10.13039/501100005416Research Council of Norway (342628/L10) and The 10.13039/501100012111Norwegian Institute for Water Research (NIVA) internal resources for AL in her role as Key Researcher.

## Data availability statement

All data are included in the article/supplementary material or referenced in the article.

## CRediT authorship contribution statement

**Vilde K. Snekkevik:** Writing – review & editing, Writing – original draft, Visualization, Methodology, Investigation, Conceptualization. **Matthew Cole:** Writing – review & editing, Conceptualization. **Alessio Gomiero:** Writing – review & editing, Writing – original draft. **Marte Haave:** Writing – review & editing, Writing – original draft, Methodology, Investigation. **Farhan R. Khan:** Writing – review & editing, Writing – original draft, Methodology, Investigation, Conceptualization. **Amy L. Lusher:** Writing – review & editing, Writing – original draft, Resources, Project administration, Methodology, Conceptualization.

## Declaration of competing interest

The authors declare that they have no known competing financial interests or personal relationships that could have appeared to influence the work reported in this paper.

## Figures and Tables

**Table S1:** Overview of analysed articles, including their focus area, primary author, and classification as peer-reviewed or review article.

	Investigated kitchen utensil	Reference	Research or review article
Food Preparation	Cutting boards	Habib et al., 2022a	Research Article
	Chopping boards	Luo et al., 2022	Research Article
	Cutting boards	Yadav et al., 2023	Research Article
	Cutting boards	Habib et al., 2022b	Research Article
	Mixing bowls	Jander et al., 2022	Research Article
	Kitchen blender	Luo et al., 2023	Research Article
Food Storage	Food container	Hee et al., 2022	Research Article
	Food containers	Hussain et al., 2023	Research Article
Cooking Equipment	Non-stick cookware	Luo et al., 2022	Research Article
Cleaning Equipment	Cleaning sponge	Luo et al., 2022	Research Article
Food Preservation	Victuals Packaging	Guan et al., 2021	Research Article
Appliances	Kettle	Shi et al., 2022	Research Article
	Washing machine	Belzagui et al., 2019	Research Article
	Washing machine	Berruezo et al., 2021	Research Article
	Washing machine	Cai et al., 2021	Research Article
	Drying machine	O'Brien et al., 2020	Research Article
	Washing machine	Dalla Fontana et al., 2021	Research Article
	Washing machine	De Falco et al., 2020	Research Article
	Washing machine	Dreillard et al., 2022	Research Article
	Washing machine	Galvao et al., 2020	Research Article
	Washing machine	Hartline et al., 2016	Research Article
	Washing machine	Jenner et al., 2021	Research Article
	Washing machine	Lant et al., 2022	Research Article
	Washing machine	Lim et al., 2022	Research Article
	Washing machine	Palacios-Marin et al., 2022	Research Article
	Washing machine	Vassilenko et al., 2021	Research Article
