# Supplementary material for: Beyond the food on your plate: Investigating sources of microplastic contamination in home kitchens
Source: Heliyon. 2024 Jul 24;10(15):e35022. doi: 10.1016/j.heliyon.2024.e35022 (PMC11336334; doi:10.1016/j.heliyon.2024.e35022)
Supplement: Multimedia component 1 [file mmc1.docx]

SUPPLEMENTARY

Table S1. Overview of analysed articles, including their focus area, primary author, and classification as peer-reviewed or review article.

|  | **Investigated kitchen utensil** | **Reference** | **Research or review article** |
| --- | --- | --- | --- |
| Food Preparation | Cutting boards | Habib et al., 2022a | Research Article |
|  | Chopping boards | Luo et al., 2022 | Research Article |
|  | Cutting boards | Yadav et al., 2023 | Research Article |
|  | Cutting boards | Habib et al., 2022b | Research Article |
|  | Mixing bowls | Jander et al., 2022 | Research Article |
|  | Kitchen blender | Luo et al., 2023 | Research Article |
| Food Storage | Food container | Hee et al., 2022 | Research Article |
|  | Food containers | Hussain et al., 2023 | Research Article |
| Cooking Equipment | Non-stick cookware | Luo et al., 2022 | Research Article |
| Cleaning Equipment | Cleaning sponge | Luo et al., 2022 | Research Article |
| Food Preservation | Victuals Packaging | Guan et al., 2021 | Research Article |
| Appliances | Kettle | Shi et al., 2022 | Research Article |
|  | Washing machine | Belzagui, et al., 2019 | Research Article |
|  | Washing machine | Berruezo, et al., 2021 | Research Article |
|  | Washing machine | Cai et al. 2021 | Research Article |
|  | Drying machine | O’Brien et al., 2020 | Research Article |
|  | Washing machine | Dalla Fontana et al., 2021 | Research Article |
|  | Washing machine | De Falco et al., 2020 | Research Article |
|  | Washing machine | Dreillard et al., 2022 | Research Article |
|  | Washing machine | Galvao et al., 2020 | Research Article |
|  | Washing machine | Hartline et al., 2016 | Research Article |
|  | Washing machine | Jenner et al., 2021 | Research Article |
|  | Washing machine | Lant et al., 2022 | Research Article |
|  | Washing machine | Lim et al., 2022 | Research Article |
|  | Washing machine | Palacios-Marin et al., 2022 | Research Article |
|  | Washing machine | Vassilenko et al., 2021 | Research Article |
